# Azoramide, a novel regulator, favors adipogenesis against osteogenesis through inhibiting the GLP-1 receptor-PKA-β-catenin pathway

**DOI:** 10.1007/s13577-025-01192-0

**Published:** 2025-03-20

**Authors:** Zhao Yan, Banjun Ruan, Zheng Zhu, Xiaorui Cao, Zifan Lu

**Affiliations:** 1 Department of Anatomy and K.K. Leung Brain Research Centre, Fourth Military Medical University, Xi’an, 710032 China; 2 Institute of Orthopedic Surgery, Xijing Hospital, Fourth Military Medical University, Xi’an, 710032 People’s Republic of China; 3 State Key Laboratory of Cancer Biology, Department of Pharmacogenomics, Fourth Military Medical University, Xi’an, 710032 People’s Republic of China; 4 Department of Urology, Xijing Hospital, Fourth Military Medical University, Xi’an, 710032 Shanxi China; 5 Innovation Research Institute, Xijing Hospital, Airforce Medical University, Xi’an, 710032 Shanxi China

**Keywords:** Azoramide, MSC, Osteogenesis, Adipogenesis, Glucagon-like peptide-1 receptor, Wnt

## Abstract

The reciprocal fate decision of mesenchymal stem cells (MSCs) to either bone or adipocytes is determined by Wnt-related signaling and the glucagon-like peptide-1 receptor (GLP-1R). Azoramide, an ER stress alleviator, was reported to have an antidiabetic effect. In this study, we investigated the function of azoramide in regulating the lineage determination of MSCs for either adipogenic or osteogenic differentiation. Microcomputed tomography and histological analysis on bone morphogenetic protein (BMP)2-induced parietal periosteum bone formation assays, C3H10T1/2 and mouse bone marrow MSC-derived bone formation and adipogenesis assays, and specific staining for bone tissue and lipid droplets were used to evaluate the role of azoramide on the lineage determination of MSC differentiation. Cells were harvested for Western blot and quantitative real-time polymerase chain reaction (PCR), and immunofluorescence staining was used to explore the potential mechanism of azoramide for regulating MSC differentiation. Based on MSC-derived bone formation assays both in vivo and in vitro, azoramide treatment displayed a cell fate determining ability in favor of adipogenesis over osteogenesis. Further mechanistic characterizations disclosed that both the GLP-1R agonist peptide exendin-4 (Ex-4) and GLP-1R small interfering (si)RNA abrogated azoramide dual effects. Moreover, cAMP-protein kinase A (PKA)-mediated nuclear β-catenin activity was responsible for the negative function of azoramide on bone formation in favor of adipogenesis. These data provide the first evidence to show that azoramide may serve as an inhibitor against GLP-1R in MSC lineage determination.

**Supplementary Information:**

The online version contains supplementary material available at 10.1007/s13577-025-01192-0.

## Background

Mesenchymal stem cells (MSCs) are nonhemopoietic stem cell progenitors found in bone marrow and many other tissues. They have multiple differentiation potentials, such as to bone and fat [1, 2]. The lineage differentiation directions are regulated by various signaling pathways including Wnt, bone morphogenetic protein (BMP), and Notch, among others [3]. Moreover, both in vitro and in vivo studies have shown that a reciprocal fate decision is frequently observed between adipogenesis and osteogenesis.

Increasing evidence suggests that Wnt signaling may have an important role in regulating MSC differentiation [4]. The activation of Wnt signaling has been reported to facilitate osteogenic differentiation while inhibiting adipogenic differentiation of MSCs [5, 6].

As previously shown, there are two major types of Wnt signaling: canonical Wnt and noncanonical Wnt pathways [7]. The canonical Wnt ligands, such as Wnt10b, could stabilize β-catenin by preventing its phosphorylation. Non-phosphorylated β-catenin translocates into the nucleus and regulates differentiation-related target gene expressions; genetic deletion of β-catenin in embryonic mesenchymal progenitors abolishes mature osteoblast generation. In addition, loss of β-catenin in the mesenchyme of the developing mouse uterus was found to be a switch to adipogenesis in the myometrium [8]. Wnt10b suppresses the expression of the adipogenic transcription factors peroxisome proliferator-activated receptor (PPAR)γ and CCAAT/ enhancer-binding protein-α (C/EBPα) in a β-catenin- and T-cell factor (TCF)-dependent manner. With the aging progress, Wnt10b reduction causes an increase in adipocytes at the expense of bone loss [9]. This differentiation defect may contribute to aging-related disease.

There are many other regulators besides Wnt signaling involved in MSC-related differentiation homeostasis. Glucose-dependent insulinotropic polypeptide (GIP) and glucagon-like peptide-1 (GLP-1) exert their beneficial effects on diabetes through distinct G protein-coupled receptors, which are highly expressed on islet β cells [10]. The production of cAMP and influx of Ca2 + are vital components in the biosynthesis and secretion of insulin. The GLP-1 and GIP receptors are also widely expressed in non-islet cells. Many previous reports indicated that they caused a positive effect on bone metabolism. Recently, some new experiments have provided direct evidence to show that the presence of GLP-1 receptors (GLP-1R) in MSCs is required for the bone-inducing effects of MSC in response to exendin-4 (Ex-4), an agonist of GLP-1R. The downstream pathways were regulated by cAMP-protein kinase A (PKA)-mediated activation of nuclear β-catenin [11, 12]. Recently, a new compound, azoramide, has been defined as a potential antidiabetic drug candidate [13]; it exhibited potent antidiabetic efficacy by improving peripheral insulin sensitivity and pancreatic β-cell function. While azoramide is a widely used antidiabetic drug, its effect on bone health remains largely unexplored. To investigate this, we conducted in vivo experiments to assess its impact on ectopic bone formation. Interestingly, our results indicated that azoramide treatment led to a decrease in bone formation, prompting us to design further experiments. Our research focuses on the differentiation of mesenchymal stem cells (MSCs) and the identification of novel targets to modulate this process. The Wnt/β-catenin pathway has long been recognized as a key regulator of MSC differentiation. Moreover, numerous studies have demonstrated that GLP-1 receptor (GLP-1R) agonists can influence cellular activities across various cell types through the β-catenin pathway. In our preliminary experiments, we observed that azoramide inhibited ectopic bone formation. Given the significant role of β-catenin in MSC differentiation, we sought to further investigate the impact of azoramide—an antidiabetic drug and GLP-1R inhibitor—on MSC differentiation. The underlying mechanisms were related to antagonism of GLP-1R-mediated signaling.

## Methods

### Animals

C57BL/6 mice were purchased from the Experimental Animal Center of the Fourth Military Medical University. The mice were housed at the animal care facility at 22 °C with 12 h light/dark cycles. In vivo analysis of azoramide responses by BMP2 calvarial injection was conducted as described below. Briefly, 14 day-old mice were injected with recombinant BMP2 (10 mg/ml; Wish biotechnology, Beijing, China) 30 μl per injection, three times a day for 5 days, into the periosteal tissue overlying the right parietal bone; saline vehicle control was injected into the left parietal bone. Meanwhile, azoramide compound (20 mg/ml, 30 μl per injection; MedChem Express, Princeton, NJ, USA) was administered via intraperitoneal injection once a day for 14 consecutive days. Animals were then sacrificed after the last injection to analyze bone formation. All animal care and experimental procedures were performed under approval of the Institutional Animal Care and Use Committee of the Fourth Military Medical University (Approval No. 2016013).

### Cell culture and differentiation

The C3H10T1/2 cell line was obtained from the Center for Type Culture Collection of China. Cells were cultured in complete medium containing Dulbecco’s modified Eagle’s medium (DMEM; Gibco, Gaithersburg, MD, USA) with 10% fetal bovine serum (FBS; Gibco) and penicillin/ streptomycin (Invitrogen, Carlsbad, CA, USA), and used for the in vitro experiments. To induce osteogenic differentiation, cells were cultured in osteogenic media containing 100 nM dexamethasone (Sigma-Aldrich, St. Louis, MO, USA), 50 μg/ml ascorbic acid (Sigma-Aldrich), and 10 mM β-glycerophosphate (Sigma-Aldrich). To induce adipogenic differentiation, cells were cultured in adipogenic medium containing 10 μg/ml insulin (Sigma- Aldrich), 1 μM dexamethasone (Sigma-Aldrich), 0.5 mM 3-isobutyl-1-methylxanthine (Sigma-Aldrich), and 1 μM rosiglitazone (Sigma-Aldrich). All cell-based in vitro experiments were repeated three times.

### Transfection

Two GLP-1R target sequences (si-glp-1r-1: 5′-CCGGA CCTTTGATGACTAT-3′; si-glp-1r-1: 5′-GGAACTAC ATCCACCTGAA-3′) were designed to synthesize small interfering (si)RNA by Sangon (Shanghai, China). A scrambled siRNA sequence (sense: 5′-UUCUCCGAACGUGUCACGUTT- 3′ and antisense: 5′- ACGUGACAC GUUCGGAGAATT-3′) was used as a control. Cells were transfected with Lipofectamine^™^ 2000 Transfection Reagent (Invitrogen) as described in the manufacturer’s protocol. Cells were plated in six-well plates starting with cell density at > 90%. Four hours before transfection, the medium was changed to Opti-MEM^®^ Medium (Gibco). The siRNA (100 nM) was incubated with 5 μl Lipofectamin^™^ 2000 in Opti-MEM^®^ Medium for 20 min at room temperature before transfection. Then, 6 h later, the medium was switched to complete culture medium and, 48 h before induction, C3H10T1/2 cells were transfected with scramble or GLP-1R siRNA. The expression plasmid harboring with green fluorescent protein (GFP) was used as an indicator for transfection efficiency. Cells transfected with scramble or GLP-1R siRNA were used for osteogenic and adipogenic differentiation.

### Microcomputed tomography and histology

Dissected calvaria from BMP2-injected mice were fixed immediately in 70% ethanol and scanned with a microcomputed tomography (micro-CT) system (Inveon Micro-CT, Germany). Image acquisition was performed at 80 kV and 500 μA with a resolution of 2048 × 2048 pixels and a voxel size of 10.56 μm. Quantitation of heterotopic new bone formation and reconstruction into three dimensional (3D) volumes was made using software (Inveon Research Workplace 2.2 and Inveon Acquisition Workplace, version 1.4.3.6) that was compatible with the micro-CT system. For histology analysis, dissected calvaria were fixed in 4% paraformaldehyde, and decalcified tissues were embedded into paraffin accordingly. Sections of the embedded bone tissues were subjected to hematoxylin and eosin (H&E) staining to count the osteoblasts and toluidine blue staining to visualize adipocytes. Quantitative analysis of histological staining was performed with ImageJ, as previously described (NIH, Bethesda, MD, USA) [14].

### Alkaline phosphatase staining

Mouse bone marrow-derived MSCs were developed as reported. Mouse MSC and C3H10T1/2 cells were seeded in six-well plates and cultured for 7 days in osteogenic differentiation medium with or without azoramide. The cells were then rinsed three times with phosphate buffered saline (PBS), fixed in 4% paraformaldehyde at room temperature for 15 min, and washed three more times with PBS. For staining, an alkaline phosphatase (ALP) substrate solution (Beyotime, Shanghai, China) was added to the fixed cells for 30 min at room temperature. Cells were then washed three times with distilled water, and images were photographed.

### Quantification of ALP activity

After MSC or C3H10T1/2 cells were cultured in osteogenic differentiation medium with different treatments for 7 days, the cells were lysed and ALP activity was assayed with the Beyotime Alkaline Phosphatase Assay Kit, calculated after normalization to the total protein content according to the protocol from the supplier [15].

### Alizarin red S staining and quantification

MSC and C3H10T1/2 cells were cultured in osteogenic differentiation medium for 21 days. Alizarin red S staining was used to measure the degree of mineralization of cells on the sample surfaces with 1% Alizarin red S staining solution (Sigma-Aldrich) for 30 min at room temperature. Cells were then washed with distilled water three times to remove unbound dye and photographed. For quantification of mineralization, the staining was solubilized with 100 mM acetyl pyridinium chloride (Sigma-Aldrich) and the extracted stains were then measured using a microplate absorbance reader (Bio-Rad, Hercules, CA, USA) at 562 nm to quantify the osteogenic differentiation of C3H10T1/2 cells [16].

### Oil red O staining and quantification

For Oil red O staining, MSC and C3H10T1/2 cells were cultured in six-well culture plates for 8 days. Cells were washed with PBS, fixed in 4% paraformaldehyde for 15 min at room temperature, and then washed with 60% isopropanol. The cells were stained with 0.6% (w/v) Oil red O (Sigma-Aldrich) solution (60% isopropanol, 40% water) for 30 min at room temperature. Cells were then washed with distilled water three times to remove unbound dye and photographed. Stained Oil red O was also eluted with 100% isopropanol (v/v) and quantified by measuring the optical absorbance at 540 nm [17].

### Real-time polymerase chain reaction

Following various treatments, cells were washed with ice cold PBS and subjected to RNA extraction with TRIzol^®^ Reagent (Invitrogen); RNA samples (2 μg) were reversetranscribed into complementary DNA (cDNA) using MMLV Reverse Transcriptase (Invitrogen). cDNA was then diluted and used for quantification (with β-actin gene as a control) by real-time quantitative polymerase chain reaction (qPCR), which was performed using the CFX96 Real- Time PCR system (Bio-Rad) with the Power SYBR Green PCR Master Mix (Takara, Tokyo, Japan). The primer pairs used for amplification in this study were: Runt-related transcription factor 2 (Runx2): 5′-CCACCTCTGACT TCTGCCTC-3′ (forward) and 5′-ATGAAATGCTTGGGAACTGC- 3′ (reverse); Sp7: 5′-CCCTTCTCAAGCACC AATGG-3′ (forward) and 5′-AGGGTGGGTAGTCATT TGCATAG-3′ (reverse); Integrin binding sialoprotein (Ibsp): 5′-CGGCCACGCTACTTTCTTTA-3′ (forward) and 5′-TTGAAGTCTCCTCTTCCTCCC-3′ (reverse); bone gamma-carboxyglutamate protein (Bglap): 5′-GGCT TAAAGACCGCCTACAG-3′ (forward) and 5′-GAGAG GACAGGGAGGATCAA-3′ (reverse); PPARγ: 5′-GGTC TCGGTTGAGGGGAC-3′ (forward) and 5′-CCATGG TAATTTCAGTAAAGGGTAG-3′ (reverse); Fatty acid binding protein 4 (Fabp4): 5′-CGCAGACGACAGGA AGGTGAA-3′ (forward) and 5′-GAAGTCACGCCTTT CATAACACAT-3′ (reverse); Adiponectin: 5′-CAGTG GATCTGACGACACCAA-3′ (forward) and 5′-CGTCAT CTTCGGCATGACTG-3′ (reverse); Tcf7l2: 5′-CCCATC CGCTAGGATGGTTAG-3′ (forward) and 5′-TGGGGTAGGGGTGTCTGAAT-3′ (reverse); β-actin: 5′-AGCGGGAAATCGTGCGTGAC-3′ (forward) and 5′-TGGAAGGTGGACAGCGAGGC-3′ (reverse).

### Western blotting

Cells cultured under the different conditions were washed with ice-cold PBS and lysed in RIPA buffer containing a protease inhibitor cocktail (Roche, Mannheim, Germany). Protein concentrations were determined using the BCA assay kit (Thermo Scientific, Waltham, MA, USA). Total protein of 50 μg was separated on 12% SDSpolyacrylamide gels and transferred to nitrocellulose membrane (Millipore, Billerica, MA, USA), and the membrane was blocked with 5% nonfat milk in Tris-buffered saline (TBS). The membrane was then incubated with the appropriate primary antibodies in TBS at 4 °C overnight. Antibodies for Runx2, PPARγ, Fabp4, β-catenin, and phospho-β-catenin were from Cell Signaling Technology (Beverly, MA, USA). GLP-1R antibody was from Ruiying Biological (Suzhou, Jiangsu, China), and antibodies against PKAc and β-actin were from Sangon. Secondary antibodies conjugated to IRDye 800 were detected using an Odyssey infrared imaging system (LI-COR, Lincoln, NE, USA). All Western blots were independently replicated at least three times and the intensities of the bands were quantified using ImageJ software.

### Immunofluorescence staining

C3H10T1/2 cells were seeded on coverslips. After different treatment, cells were fixed with 4% paraformaldehyde for 15 min and permeabilized with 0.1% Triton X-100 in TBS for 15 min. After being blocked in 5% FBS in PBS for 30 min, cells were incubated with anti-β-catenin (Cell Signaling Technology) overnight at 4 °C. After washing three times with PBS, cells were incubated with DyLight^™^ 594-conjugated secondary antibodies (Thermo Scientific) for 60 min at room temperature and then stained with 4′,6-diamidino-2-phenylindole (DAPI; Sigma-Aldrich) for 10 min. Slides were then washed three times and mounted. Immunofluorescence was detected using an Olympus inverted fluorescence microscope.

### Statistical analysis

All data are shown as the mean ± standard deviation (SD). Data were analyzed using either a one-way analysis of variance (ANOVA) followed by a Tukey’s post-hoc test for comparison of multiple groups or an independent Student’s t test for comparison of two groups, as described in the individual figure legends (GraphPad Prism 6.0 software). A value of p < 0.05 was considered statistically significant.

## Results

### Azoramide impairs BMP2-induced heterotopic new bone formation on the parietal bone periosteum

**Fig. 1:**
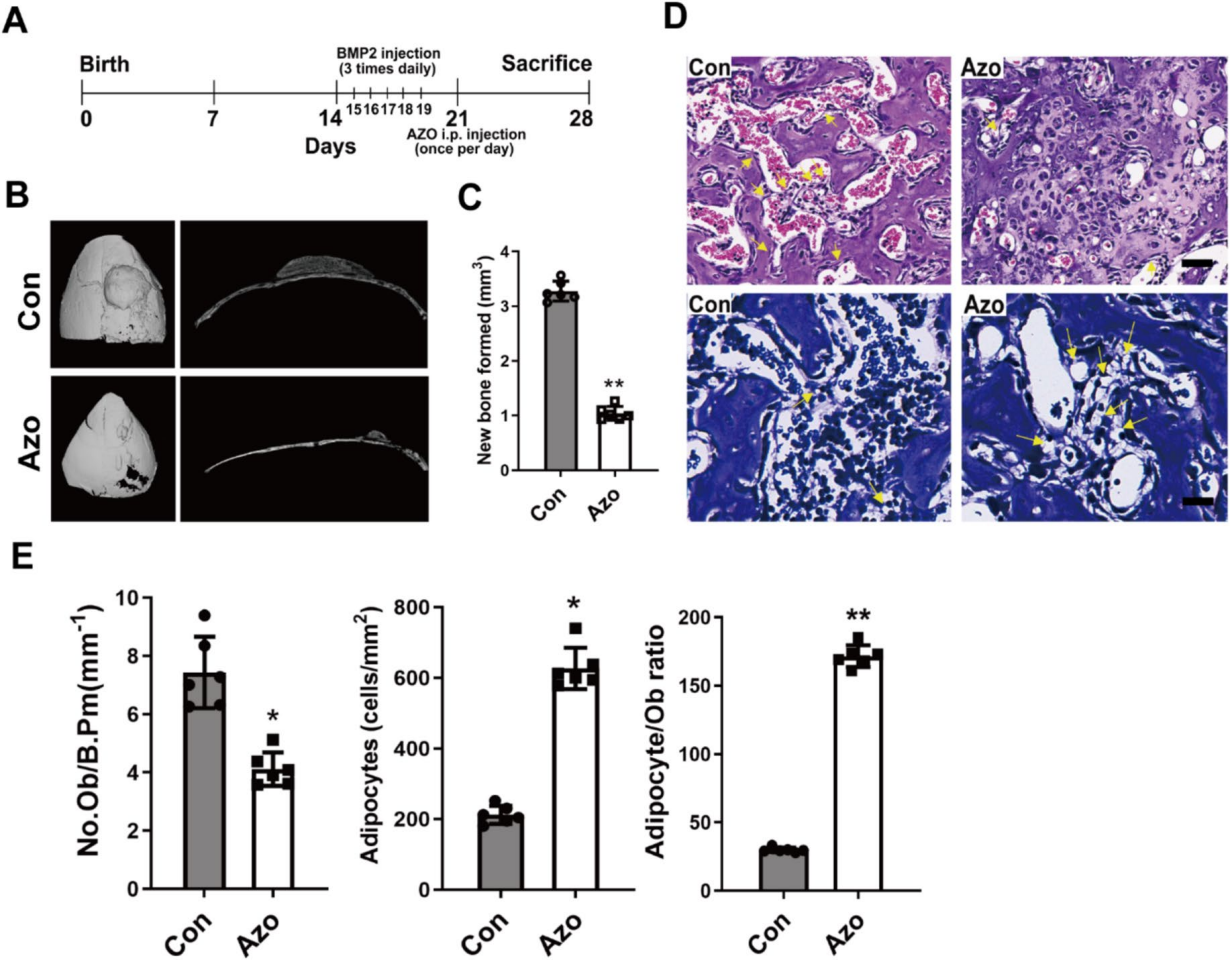
Reduced BMP2-induced bone formation in azoramide-treated mice. **A** Schematic of a bone morphogenetic protein (BMP)2 calvarial injection model. **B** Micro-CT 3D reconstruction and cross-sectional image of control (Con) and Azoramide-treated (Azo) calvarium (red arrows) following treatment with BMP2 (*n* = 6 biologically independent samples in each group). **C** micro-CT quantification of newly formed bone in response to BMP2. **D** Representative hematoxylin and eosin staining to count osteoblasts, and toluidine blue staining to visualize adipocytes (yellow arrows) of BMP-induced newly formed bone from control (Con) and Azoramide-treated (Azo) mice. Scale bar = 100 μm. *E* Histomorphometric quantification of osteoblast numbers (N.Ob) per bone perimeter (B.Pm) and adipocyte numbers. *N* = 3 biologically independent experiments; *p < 0.05, **p < 0.01, versus control mice. i.p. intraperitoneal

To examine the in vivo effect of azoramide on bone formation, a model of BMP2-induced heterotopic new bone formation on the parietal bone was developed [18]. Subcutaneous injection of BMP2 on the progenitor-rich periosteum lining of the calvaria induces the local recruitment and differentiation of precursors into osteoblasts, resulting in the de novo formation of cancellous bone and bone marrow [19]. Briefly, 14-day-old C57BL/ 6 mice were injected with recombinant BMP2 three times a day for 5 days into the periosteal tissue overlying the right parietal bone with vehicle injected to the left parietal bone as a negative control. Meanwhile, azoramide and DMSO control were administered via intraperitoneal injection once a day for 14 consecutive days (Fig. 1a). The azoramide treatment group, induced by BMP2, showed remarkable reductions in new bone formations compared with the control group (Fig. 1b); the volume of newly formed bone was approximately 68% smaller in the groups injected with azoramide (Fig. 1b, c). These data indicated that azoramide treatment could impair the BMP2-induced formation of new bone. Histological analysis of the local new bone tissues in responses to azoramide showed an obvious decrease in the number of osteoblasts and a significant increase in the number of adipocytes in the newly formed bone marrow spaces after azoramide treatment (Fig. 1d, e). Taken together, the results suggested that azoramide inhibited the bone forming ability of mesenchymal progenitor cells under BMP2 induction. More likely, it preferentially induces MSCs towards adipocytes rather than osteoblasts.

### *Azoramide inhibits the osteogenic differentiation while enhancing the adipogenic potential of C3H10T1/2 cells and mouse MSCs *in vitro

**Fig. 2:**
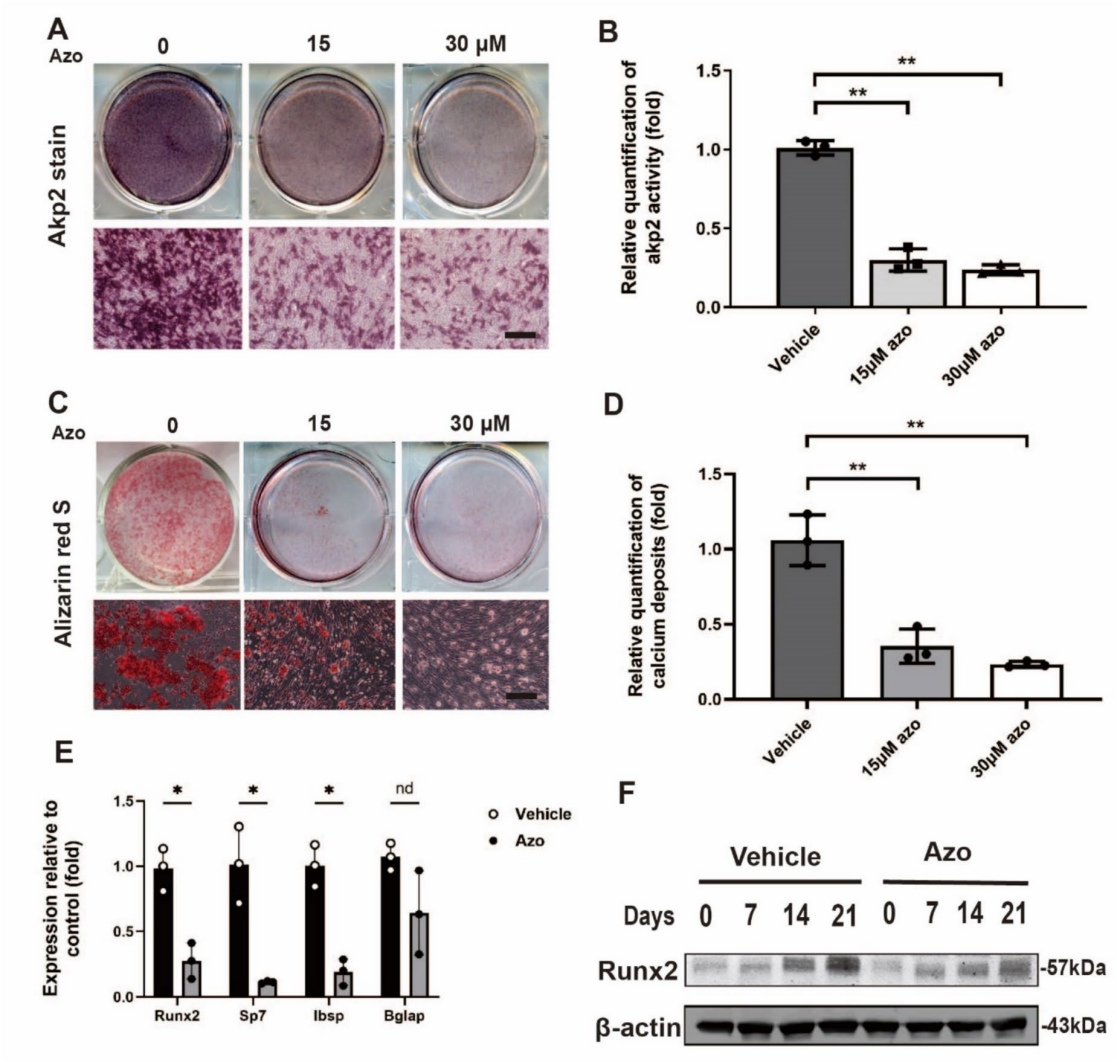
Inhibited osteogenic differentiation potential of C3H10T1/2 cells with azoramide treatment in vitro. **A**, **B** Alkaline phosphatase (ALP) staining and quantification of ALP activity of cellular extracts subjected to different concentrations of azoramide (Azo). Scale bar = 100 μm. **C**, **D** Alizarin red S staining for mineral deposition and its quantification with different concentrations of azoramide. Scale bar = 100 μm. Images are representatives of three independent experiments with biological duplicates. **E** Real-time qPCR of Runt-related transcription factor 2 (Runx2), Sp7, integrin binding sialoprotein (Ibsp), and bone gamma-carboxyglutamate protein (Bglap) mRNA in C3H10T1/2 cells subjected to 15 μM azoramide. f Western blot of Runx2 protein in C3H10T1/2 cells subjected to 15 μM azoramide. *n* = 3 biologically independent experiments. *p < 0.05, **p < 0.01, azoramide versus vehicle

To investigate the role of azoramide in osteogenesis, C3H10T1/2 cells were cultured in osteogenesis induction medium with different concentrations of azoramide. ALP staining and quantification of ALP activity showed that azoramide remarkably inhibited the osteogenic differentiation (Fig. 2a, b). Alizarin red S staining of the mineralized nodule, an indicator of bone formation, demonstrated that azoramide impaired mineral depositions at day 21 in a dose-dependent manner (Fig. 2c, d). Moreover, our results revealed that azoramide significantly decreased the expression of osteoblast differentiation-related genes and proteins, such as Runx2 and Sp7 transcription factor (Sp7) at day 7. It also decreased the expression of mature matrix-producing osteoblast marker genes, such as Ibsp and Bglap by realtime qPCR at day 14 (Fig. 2e, f).

**Fig. 3:**
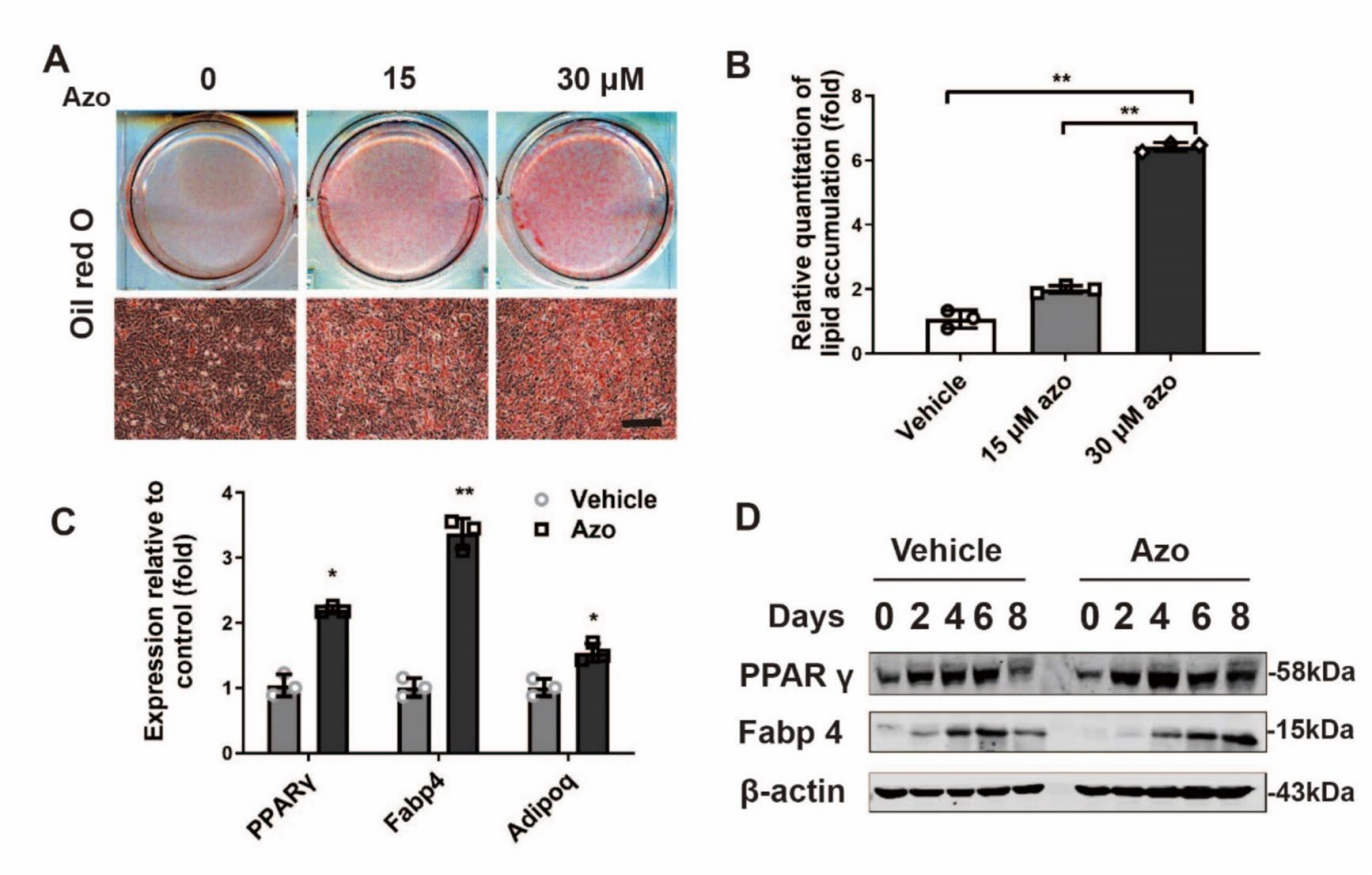
Enhanced adipogenic differentiation potential of C3H10T1/2 cells and mouse-derived MSCs with azoramide treatment in vitro. **A**, **B** Oil red O staining for lipid production and its quantification with different concentrations of azoramide (Azo). Scale bar = 100 μm. Images are representatives of three independent experiments with biological duplicates. **C** Real-time qPCR of peroxisome proliferator-activated receptor gamma (PPARγ), fatty acid binding protein 4 (Fabp4), and adiponectin (Adipoq) mRNA in C3H10T1/2 cells subjected to 15 μM azoramide. **D** Western blot of PPARγ and Fabp4 proteins in C3H10T1/2 cells subjected to 15 μM azoramide. Scale bar = 100 μm. *p < 0.05, **p < 0.01, azoramide versus vehicle. *n* = 3 biologically independent experiments. Bglap bone gamma-carboxyglutamate protein, Ibsp integrin binding sialoprotein, Runx2 Runt-related transcription factor 2

We then evaluated the potential of C3H10T1/2 cells to differentiate into mature adipocytes under azoramide treatment. Intriguingly, there was increased lipid production in the adipogenesis process under azoramide, as shown by Oil red O staining at day 8 (Fig. 3a, b). Consistently, mRNA and protein levels of PPARγ at day 3 and Fabp4 and adiponectin mRNA expression at day 8 were all increased (Fig. 3c, d).

To further confirm the above results, we investigated mouse MSCs as a differentiation cell model and observed that azoramide enhanced MSCs to adipogenesis; meanwhile it prohibited osteogenesis in a dose-dependent manner, based on the results of lipid droplets stains with Oil red O and ALP stains for osteogenesis. Moreover, specific mRNA changes in lineage differentiations markers were also seen. All the data from the bone marrow MSCs are consistent with the C3H10T1/2 cell-derived results.

To sum up, the above in vivo and in vitro studies support that azoramide directly induces C3H10T1/2 and mouse MSCs to adipogenic differentiation rather than osteoblast induction.

### Ex-4, an agonist of GLP-1R, attenuats the effects of azoramide on C3H10T1/2 cell dual differentiation

To explore the molecular basis of the inhibitory efficiency of azoramide we used Autodock software to predict the binding mode of azoramide with GLP-1R as shown in (Additional file 1). The results revealed that azoramide showed a similar binding mode to the ligand from the crystal structure of GLP-1R (PDBID:3C5T) [20], and fitted well in the cofactor binding pocket (Additional files 1 and 2). Further interaction analysis indicated that azoramide binds tighter than the ligand in the x-ray structure. Two H-bonds were found between azoramide and GLP-1R (azoramide:N1 to ASN82:OD1 and azoramide:O1 to TYR101:OH). A π–π cation interaction was found between the Phe ring of azoramide and the Phe ring of PHE80 (Additional file 2). This might stabilize azoramide in the hydrophobic groove made by TRP120 and the nearby residues [21].

**Fig. 4:**
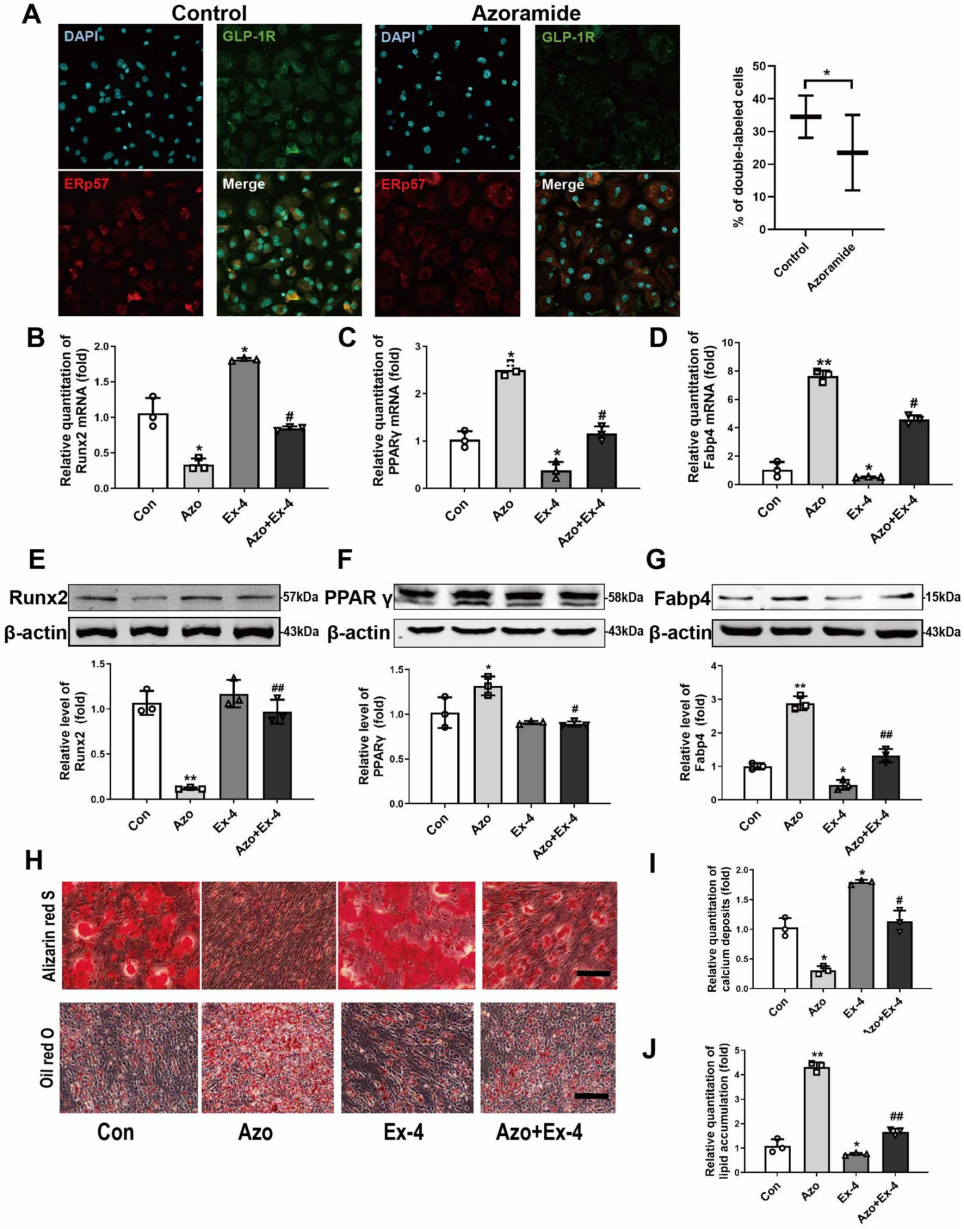
Ex-4 treatment attenuated azoramide effects on suppressing C3H10T1/2 cell osteoblast differentiation and promoting their differentiation into adipocytes. **A** Immunofluroscence of GLP-1R and ERp57 in C3H10T1/2 cells after azoramide treatment. **B**–**D** Real-time qPCR of Runt-related transcription factor 2 (Runx2), peroxisome proliferator-activated receptor gamma (PPARγ), and fatty acid binding protein 4 (Fabp4) mRNA in C3H10T1/2 cells subjected to different treatments. **E**–**G** Western blot of Runx2, PPARγ, and Fabp4 proteins in C3H10T1/2 cells subjected to different treatments. Bars represent the protein quantitative data normalized to β-actin. **H**, **I** Alizarin red S for mineral deposition and its quantification. Scale bar = 100 μm. **J**, **K** Oil red O staining for lipid production and its quantification. Scale bar = 100 μm. C3H10T1/2 cells were treated with vehicle (Con), 15 μM azoramide alone (Azo), 10 nM exendin-4 alone (Ex-4), and pretreated with azoramide followed by Ex-4 (Azo + Ex-4). Images are representatives of three independent experiments with biological duplicates. *p < 0.05, **p < 0.01, versus Con; #*p* < 0.05, ##*p* < 0.01, versus Ex-4

Following the prediction, we tested whether azoramide exerts its dual differentiation effects on C3H10T1/2 cells via GLP-1R-related signals. Since exendin-4 (Ex-4; 10 nM) acts as an agonist of GLP-1R, it was given to the cells while incubating with azoramide. The results indicated that GLP-1R activation by Ex-4 reduced the effect of azoramide on the downregulation of Runx2; meanwhile, it attenuated the effect of azoramide on the upregulation of PPARγ and Fabp4 at the mRNA and protein levels (Fig. 4a–g). Moreover, the inhibitory effects of azoramide on mineralization of differentiated osteoblasts were reversed by Ex-4 treatment, and the promoting effects of azoramide on lipid production were suppressed (Fig. 4h–j). These data indicated that azoramide may influence C3H10T1/2 cell differentiation through regulating the GLP-1R-mediated pathway.

### GLP-1R silencing abolishes the azoramide regulatory effects on C3H10T1/2 cell differentiation

**Fig. 5:**
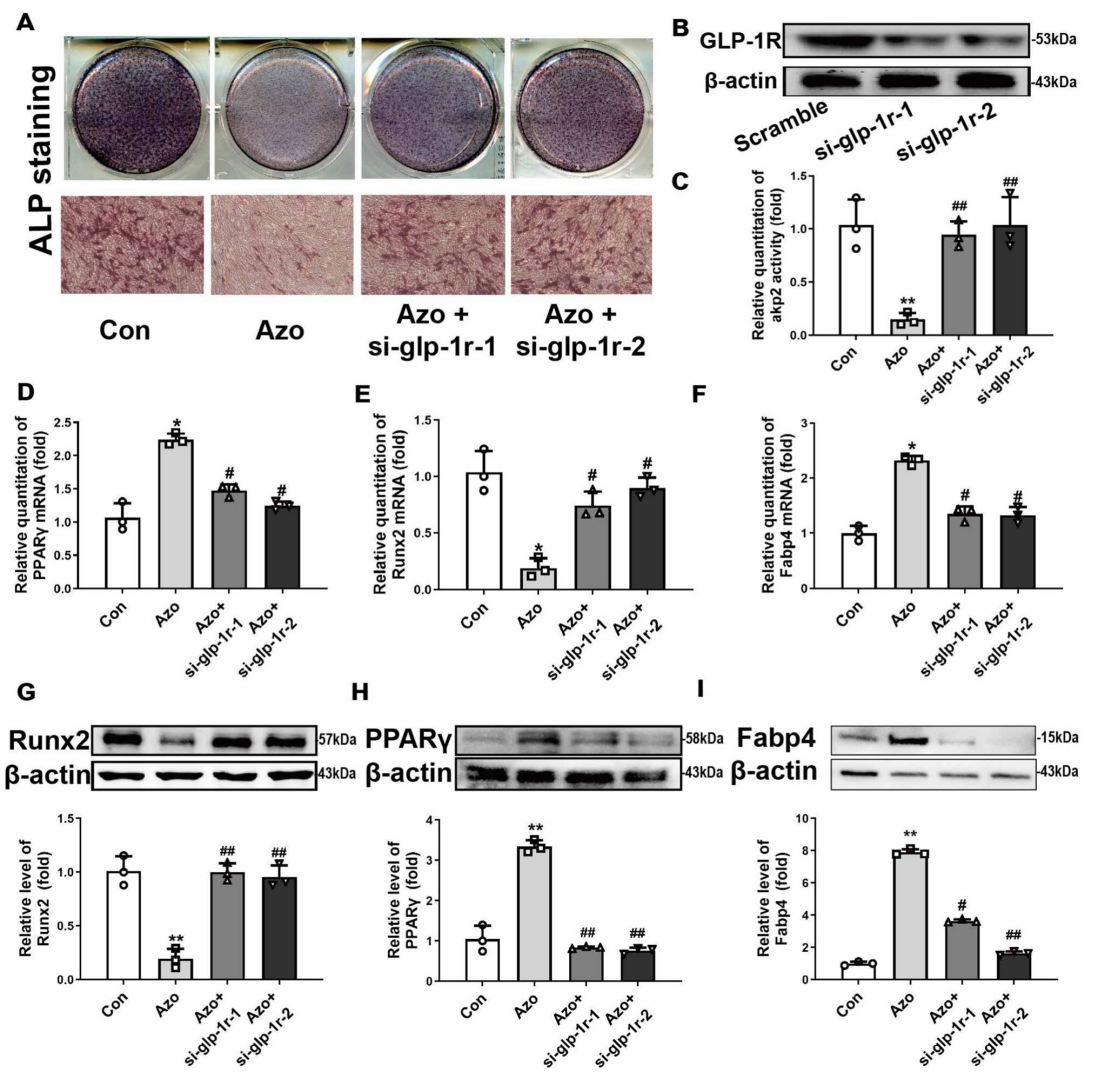
GLP-1R silencing abolished the azoramide (Azo) regulatory effects of suppressing C3H10T1/2 cell osteoblast differentiation and promoting their differentiation into adipocytes. **A**–**C** Alkaline phosphatase (ALP) staining and quantification subject to azoramide with or without glucagonlike peptide-1 receptor (GLP-1R) knock-down. Scale bar = 100 μm. **D**–**F** Real-time qPCR of Runt-related transcription factor 2 (Runx2), peroxisome proliferator-activated receptor gamma (PPARγ), and fatty acid binding protein 4 (Fabp4) mRNA in C3H10T1/2 cells subjected to different treatments. **G**–**I** Western blot of Runx2, PPARγ, and Fabp4 proteins in C3H10T1/2 cells subjected to different treatments. Bars represent the protein quantitative data normalized to β-actin. *n* = 3 biologically independent experiments. *p < 0.05, **p < 0.01, versus control (Con); #*p* < 0.05, ##*p* < 0.01, versus Azo. si small interfering. Images are representatives of three independent experiments with biological duplicates

To further validate whether azoramide mediated C3H10T1/2 cell differentiation via targeting of GLP-1R, the GLP-1R in C3H10T1/2 cells was knocked down by siRNA (Fig. 5b). The results showed that ablation of GLP- 1R abolished the inhibitory effects of osteoblast differentiation by azoramide, including changes in ALP staining and Runx2 expression (Fig. 5a, d, g). Moreover, the promoting effects of azoramide on adipogenic differentiation were also lost after GLP-1R knock-down (Fig. 5a, b). These results further supported that GLP-1R was the potential target protein responsible for azoramide-induced dual effects on C3H10T1/2 differentiation.

### Azoramide decreases the level of PKA through GLP-1R

**Fig. 6:**
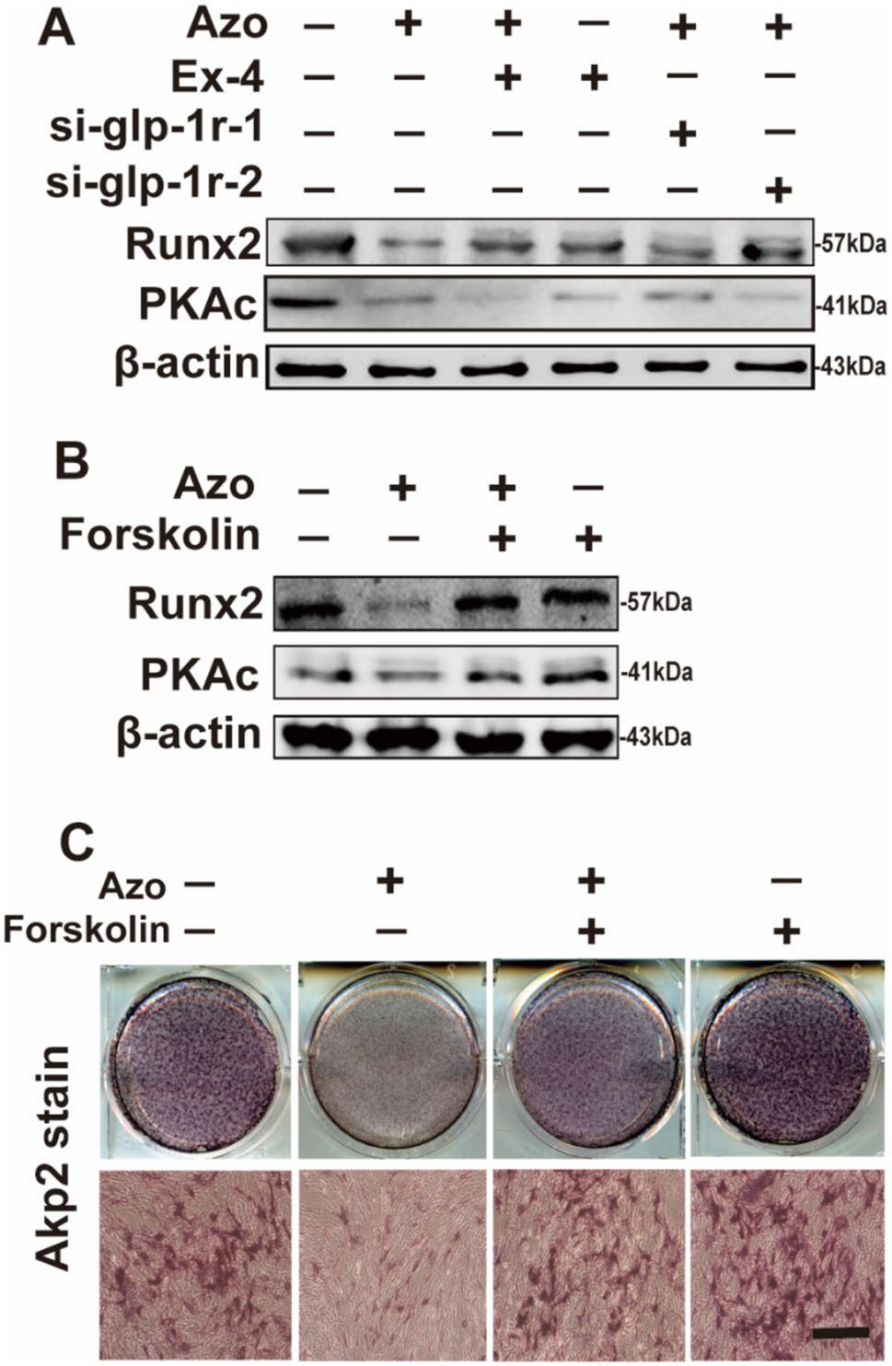
Decreased expression levels of protein kinase A (PKA) with azoramide (Azo) treatment. **A**, **B** Western blot of Runt-related transcription factor 2 (Runx2) and PKAc proteins in C3H10T1/2 cells subjected to different treatments. **C** Alkaline phosphatase (ALP) staining of cells pretreated with azoramide followed by Forskolin treatment. Scale bar = 100 μm. Images are representatives of three independent experiments with biological duplicates

To elucidate the intracellular signaling pathways of GLP-1R in C3H10T1/2 differentiation after azoramide treatment, we investigated the cAMP-mediated PKA level. Azoramide significantly decreased the expression level of the catalytic subunit (PKAc) of PKA, and the inhibition of PKAc by azoramide was abrogated by Ex-4 or GLP-1R siRNA, respectively (Fig. 6a). Moreover, forskolin, an agonist of adenylyl cyclase which is used to increase intracellular levels of cAMP, competitively enhanced the expression of PKAc inhibited by azoramide (Fig. 6b). Strikingly, the inhibitory effects of azoramide on ALP activity were reversed by forskolin treatment (Fig. 6c). These data suggested that GLP-1R-cAMP-PKA signaling pathways are responsible for mediating azoramide effects on C3H10T1/2 differentiation.

### Azoramide inhibites β-catenin nuclear translocation

**Fig. 7:**
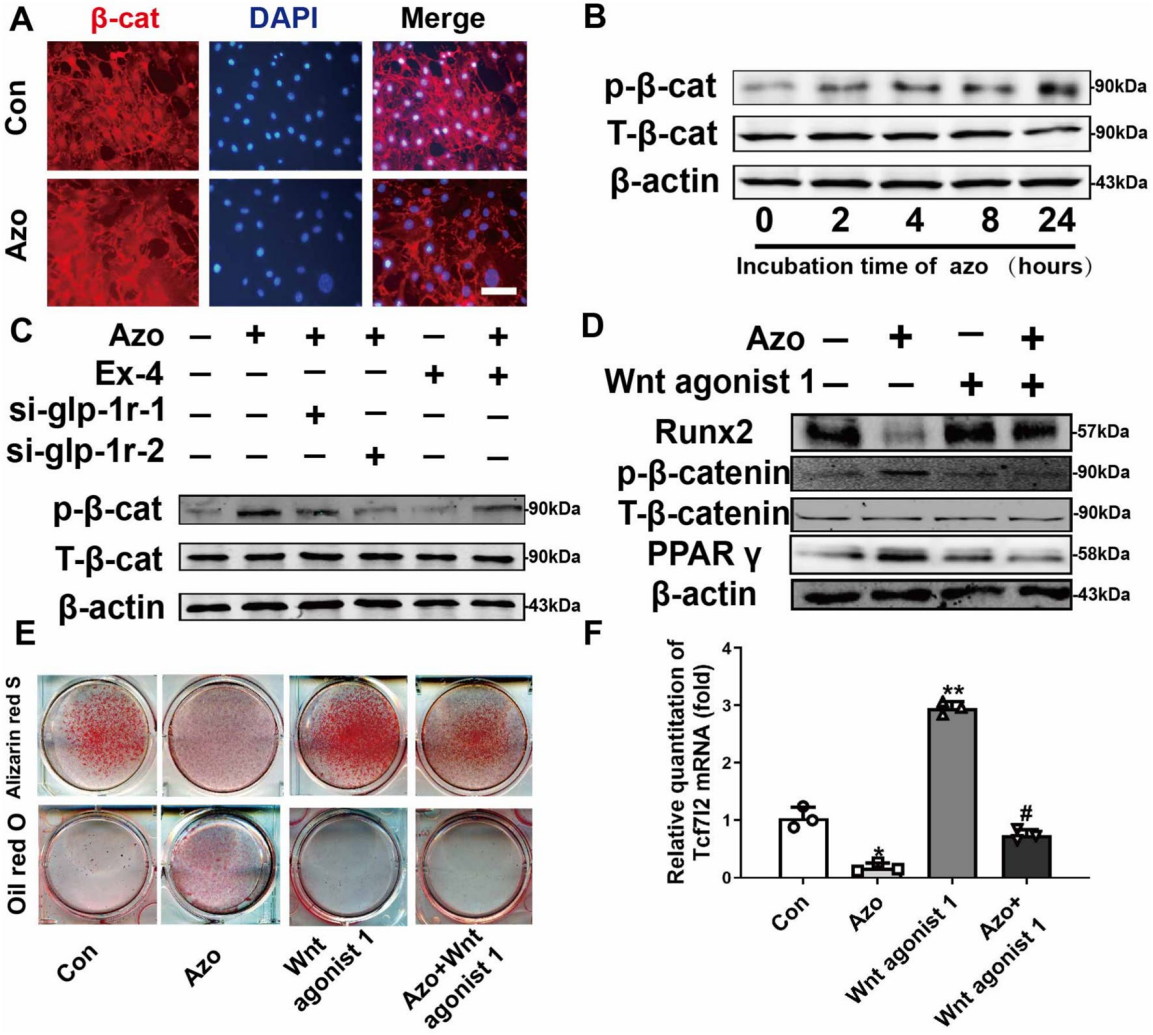
Azoramide inhibited β-catenin nuclear translocation. **A** Azoramide (Azo) treatment inhibited β-catenin nuclear localization. C3H10T1/2 cells were pretreated with osteogenic differentiation medium for 12 h followed by azoramide treatment for 6 h and cells were then fixed for immunofluorescence with anti-β-catenin antibody. Scale bar = 20 μm. **B** Western blot of phosphorylated β-catenin in C3H10T1/2 cells stimulated by 15 μM azoramide at the indicated times. **C** Western blot of phosphorylated β-catenin in C3H10T1/2 cells subjected to different treatments. **D** Western blot of Runt-related transcription factor 2 (Runx2) and peroxisome proliferator-activated receptor gamma (PPARγ) proteins in C3H10T1/2 cells treated with azoramide and Wnt agonist 1, a small-molecule agonist of the Wnt/β-catenin signaling pathway. **E** Alizarin red S staining for mineral deposition of cells subjected to different treatments. **F** Relative level of TCF7L2 mRNA in C3H10T1/2 cells subjected to different treatments. Cells were cultured in osteogenic differentiation medium. *n* = 3 biologically independent experiments. Images are representatives of three independent experiments with biological duplicates. *p < 0.05, **p < 0.01, versus control (Con); #*p* < 0.05, versus Azo. glp-1r glucagon-like peptide-1 receptor, si small interfering

Since the canonical Wnt/β-catenin signaling pathway plays a pivotal role in the modulation of lineage determinations of C3H10T1/2 cells, we evaluated whether it was involved in the effect of azoramide through GLP-1R. The immunofluorescence staining showed that azoramide treatment inhibited the translocation of β-catenin in the nucleus (Fig. 7a), also corresponding with enhanced phosphorylated β-catenin which indicated inhibition of the canonical Wnt/β-catenin signaling pathway by azoramide (Fig. 7b). Furthermore, Ex-4 and GLP-1R are both required for the phosphorylated β-catenin regulation by azoramide (Fig. 7c).

To validate the transcriptional activity of β-catenin involved in azoramide inhibition, a Wnt agonist was applied that was known to induce Wnt/β-catenin transcriptional activation of downstream target gene TCF7L2 mRNA expression [22, 23]. Our results showed that this agonist of the canonical Wnt/β-catenin signaling pathway reversed the osteoblast inhibitory effects of azoramide, in parallel with higher levels of Runx2 as well as nuclear β-catenin (Fig. 7d, f). The results were consistent with subsequent mineral deposition assays (Fig. 7e) and molecular marker changes of differentiations. Collectively, these data highly support that azoramide inhibits β-catenin nuclear translocation through GLP-1R in C3H10T1/2 cell-derived osteoblast differentiation. Overall, GLP-1R/cAMP/PKA/β-catenin signaling pathways are contributing to the dual fate decision of azoramide on mesenchymal precursor cells (Fig. 7g).

## Discussion

As previously noted, mesenchymal stromal cells are precursor cell groups characterized by specific surface markers [24–26]. These cells are not only important for early embryonic development but also for adult organ homeostasis. Under pathological conditions, such as diabetes and aging-related osteoporosis, MSCs tend to be more adipogenic than osteogenic. Therefore, defining MSC lineage differentiation will prove to be valuable for manipulating the above pathological conditions.

In terms of MSC-derived cell differentiation, adipocyte and osteocyte lineages are commonly reported [27]. Mechanically, the reciprocal cell fate determinations of MSCs are decided by Wnt-related signaling. Canonical Wnt ligand-mediated β-catenin transcriptional activity is essential for the switch between bone and fat cells in both in vivo and vitro models [27–29]. Leptin and its receptor signaling are also involved [25]. Inositol hexakisphosphate kinase 1 (Ip6k1) was recently reported to affect age-related MSC adipogenesis over osteogenesis, leading to skeletal involution and increased marrow adiposity, respectively. Inhibition of IP6K1 helps maintain insulin sensitivity and prevents obesity whilst preserving bone integrity. Therefore, IP6K1 inhibitors were regarded as more effective insulin sensitizers due to their bone-sparing properties [30].

Here, we accidentally found a chemical, azoramide, displaying a cell fate-determining effect in BMP2- induced periosteum-derived bone formation. Compared to the control, this chemical reversed BMP2 induced bone formation to more adipogenesis in the new bone marrow area. Furthermore, the C3H10T1/2 cell line and mouse MSC differentiation models further validated the in vivo observations.

It is noteworthy that azoramide was proposed as an antidiabetic drug candidate since it not only promotes peripheral insulin sensitivity but also exerts insulinotropic efficacy due to its ability to alleviate ER stress. It affects the lineage determination of MSCs and, by using the structural features of GLP-1R as well as docking results with azoramide. Our findings showed that azoramide fitted well in the cofactor binding pocket of GLP-1R. We provide more experimental evidence to show that a GLP-like peptide, resistant to dipeptidyl peptidase-4, exendin-4, exert competitive effects to block azoramide lineage-inducing and -inhibition effects. In addition, siRNA against GLP-1R almost abolished the efficacy. This evidence emphasizes the dependence of GLP-1R for the azoramide effects on MSC lineage determination. Subsequent intracellular activation by GLP-1R was dependent on cAMP-PKA-mediated nuclear β-catenin activity. All these data indicate that azoramide may act as a novel inhibitor against GLP- 1R in C3H10T1/2 cells or mice MSCs. The underlining mechanism may be that there is a direct interaction between azoramide and GLP-1R.

It is noteworthy that azoramide is known to exert a direct insulinotropic effect on the pancreas and cell lines, considering the similar insulin-promoting effects of the GLP peptide. It seems unlikely that this chemical may serve as the antagonist of GLP-1R on islet cells. Nonetheless, we cannot exclude the further possibility that azoramide may target multiple molecules in different tissues.

## Conclusions

Overall, our experiments support a novel reciprocal regulatory function of azoramide on MSC lineage differentiation, facilitating more adipogenesis instead of bone formation. At the molecular level, this may be related to the antagonistic function of GLP-1R, and subsequent blocking effects on cAMP-PKA-induced nuclear β- catenin efficacy. Notably, azoramide application may bring with it a risk of weight gain and bone loss in prospective antidiabetic drug prescriptions.

## Supplementary Information

Below is the link to the electronic supplementary material.

Supplementary file1 (XLSX 15 KB)

## Data Availability

All data generated and/or analyzed during this study are included in the published article.

## Abbreviations

ALP: Alkaline phosphatase
Bglap: Bone gamma-carboxyglutamate protein
BMP: Bone morphogenetic protein
CT: Computed tomography
DAPI: 4′,6- DIAMIDINO-2-phenylindole
DMEM: Dulbecco’s modified Eagle’s medium
Ex- 4: Exendin-4
FBS: Fetal bovine serum
Fabp4: Fatty acid binding protein 4
GFP: Green fluorescent protein
GIP: Glucose-dependent insulinotropic polypeptide
GLP-1: Glucagon-like peptide-1
GLP-1R: Glucagon-like peptide-1 receptors
H&E: Hematoxylin and eosin
Ibsp: Integrin binding sialoprotein
MSC: Mesenchymal stem cell
PBS: Phosphate-buffered saline
PKA: Protein kinase A
PPAR: Peroxisome proliferator-activated receptor
qPCR: Quantitative polymerase chain reaction
Runx2: Runt-related transcription factor 2
SD: Standard deviation
si: Small interfering
TCF: T-cell factor
